# Management Options for Reducing the Release of Antibiotics and Antibiotic Resistance Genes to the Environment

**DOI:** 10.1289/ehp.1206446

**Published:** 2013-06-04

**Authors:** Amy Pruden, D.G. Joakim Larsson, Alejandro Amézquita, Peter Collignon, Kristian K. Brandt, David W. Graham, James M. Lazorchak, Satoru Suzuki, Peter Silley, Jason R. Snape, Edward Topp, Tong Zhang, Yong-Guan Zhu

**Affiliations:** 1 Department of Civil and Environmental Engineering, Virginia Tech, Blacksburg, Virginia, USA; 2 Institute for Biomedicine, The Sahlgrenska Academy, University of Gothenburg, Gothenburg, Sweden; 3 Unilever-Safety & Environmental Assurance Centre, Sharnbrook, United Kingdom; 4 Australian National University, Canberra, Australia; 5 Canberra Hospital, Canberra, Australia; 6 Department of Plant and Environmental Sciences, University of Copenhagen, Frederiksberg, Denmark; 7 School of Civil Engineering and Geosciences, Newcastle University, Newcastle upon Tyne, United Kingdom; 8 Office of Research and Development, U.S. Environmental Protection Agency, Cincinnati, Ohio, USA; 9 Center for Marine Environmental Studies, Ehime University, Matsuyama, Ehime, Japan; 10 MB Consult Limited, Southampton, United Kingdom; 11 University of Bradford, Bradford, United Kingdom; 12 AstraZeneca, Brixham Environmental Laboratory, Brixham, United Kingdom; 13 Agriculture and Agri-Food Canada, London, Ontario, Canada; 14 Department of Civil Engineering, University of Hong Kong, Hong Kong; 15 Key Laboratory of Urban Environment and Health, Institute of Urban Environment, Chinese Academy of Sciences, Xiamen, China

**Keywords:** agriculture, antibiotic manufacturing, antibiotic resistance, aquaculture, livestock, manure management, policy, wastewater treatment

## Abstract

Background: There is growing concern worldwide about the role of polluted soil and water environments in the development and dissemination of antibiotic resistance.

Objective: Our aim in this study was to identify management options for reducing the spread of antibiotics and antibiotic-resistance determinants via environmental pathways, with the ultimate goal of extending the useful life span of antibiotics. We also examined incentives and disincentives for action.

Methods: We focused on management options with respect to limiting agricultural sources; treatment of domestic, hospital, and industrial wastewater; and aquaculture.

Discussion: We identified several options, such as nutrient management, runoff control, and infrastructure upgrades. Where appropriate, a cross-section of examples from various regions of the world is provided. The importance of monitoring and validating effectiveness of management strategies is also highlighted. Finally, we describe a case study in Sweden that illustrates the critical role of communication to engage stakeholders and promote action.

Conclusions: Environmental releases of antibiotics and antibiotic-resistant bacteria can in many cases be reduced at little or no cost. Some management options are synergistic with existing policies and goals. The anticipated benefit is an extended useful life span for current and future antibiotics. Although risk reductions are often difficult to quantify, the severity of accelerating worldwide morbidity and mortality rates associated with antibiotic resistance strongly indicate the need for action.

## Introduction

Antibiotic resistance represents a serious and growing human health threat worldwide. In many areas of the world there are no effective antibiotic therapies available for life-threatening infections, and the pace of development of novel antibiotics is now alarmingly low (Walsh 2003). Types of medical therapy and surgery that we now take for granted (e.g., bowel surgery, hip replacements, treatment of leukemia) may soon cease to be viable because the complication rate from untreatable infections will be too high (Carlet et al. 2012).

Increasing attention is being turned toward factors that potentially contribute to antibiotic resistance outside the clinical realm. The World Health Organization (WHO 2012a) has declared that emergence of antimicrobial resistance “is a complex problem driven by many interconnected factors; single, isolated interventions have little impact.*”* However, environmental pathways of antibiotic resistance have not yet been directly addressed by the WHO. In particular, recent research has highlighted soil and water environments as recipients, reservoirs, and sources of antibiotic resistance genes (ARGs) of clinical concern (Martinez 2009; Wright 2010). Likewise, soil and water environments receive inputs of antibiotics and antimicrobials, which can serve to amplify ARGs (Chee-Sanford et al. 2009; Heuer et al. 2011). Indeed, many of the resistance factors we see in clinics today have been recruited from nonpathogenic bacteria around us (Bonomo and Szabo 2006). Here, we identify and provide an overview of potential mitigation options for minimizing the spread of antibiotics and antibiotic resistance along these pathways.

In this review we consider three critically important sources of environmental exposure to antibiotics and ARGs: *a*) terrestrial agriculture; *b*) treatment of wastewater from municipalities, pharmaceutical manufacturing, and hospitals; and *c*) aquaculture. Limiting impacts to aquatic environments is of special interest because these environments serve as a source of exposure to humans via recreational use, bathing, ingestion, and aerosol inhalation. Ideally, end points for assessing the effectiveness of management strategies should not only examine antibiotic-resistant bacteria (ARBs) but also should consider the broader impact on the ARG pool (the antibiotic resistome) (Wright 2010). This would also take into account the fact that traditional culture-based methods overlook the vast majority of environmental microbes (Pace 1997).

ARBs and ARGs are abundant in human and animal fecal material; thus, active stewardship is needed to avoid gene flow to and from environmental resistance reservoirs. Both water and land can be directly affected by the industrial, agricultural, and wastewater input of antibiotics, which impose selection pressure and enable the amplification, maintenance, and spread of ARBs. Switching to alternative biocides or animal growth promoters, such as metals, will not necessarily aid in limiting the spread of antibiotic resistance, because they can also select for antibiotic resistance through co-resistance or cross-resistance (Baker-Austin et al. 2006). In addition to end-of-pipe options, source control is key. Therefore, we discuss the rationale for use of antimicrobial compounds in humans and animals, potential advantages of limiting or managing antimicrobial use, and the overall market and policy forces that impact the feasibility of management approaches.

We recognize that estimates of exposures and risks associated with environmental pathways of resistance should be pursued to a practicable extent (Ashbolt et al. 2013). However, by the time a formalized risk assessment for environmental sources of antibiotic resistance is established, opportunities for effective action may be lost. Therefore, in this critical review we focus on identifying management options that may be put into effect immediately. Ideally, simple management practices may be identified that work synergistically with existing policies and goals, such as nutrient management, runoff control, or infrastructure upgrades.

Although antibiotic resistance is clearly a global challenge, local action is necessary to reduce its spread via the environment. Indeed, regional management regimes for agricultural and clinical use of antibiotics, together with good hygiene, have in many cases proved successful in minimizing resistance on a national basis.

## Issues and Recommendations

### Limiting Agricultural Sources

*Optimizing antibiotic use.* Agricultural usage of antibiotics represents a large proportion of the overall consumption of antibiotics worldwide, although the specific antibiotics used vary extensively among countries [Danish Integrated Antimicrobial Resistance Monitoring and Research Program (DANMAP) 2010; European Medicines Agency 2011; Sarmah et al. 2006]. Most recent estimates indicate that ≥ 70% of total antibiotics used in the United States [U.S. Food and Drug Administration (FDA) 2011] and Australia (Joint Expert Advisory Committee on Antibiotic Resistance 1999) are administered to livestock. In China, about 210 million kg of antibiotics are produced annually, and 46% of these are estimated to be used in livestock (Wang and Ma 2008). In general, uncontrolled use of antibiotics and metals is increasing in Chinese agriculture and industry, corresponding to enrichment of ARGs in the manure (Zhu et al. 2013) and affected environment, particularly in soils (Wu et al. 2010).

Limiting the use and types of antibiotics, particularly “critically important antimicrobials” [Food and Agriculture Organization of the United Nations/World Organization for Animal Health/World Health Organization (FAO/OIE/WHO) 2004; WHO 2012a], in animal production is the most direct route of controlling agricultural antibiotic release into the environment, and likely also antibiotic resistance. In some countries, regulations on dosing based on clinical efficacy are in place, and growth promoters have been banned in some cases. Importantly, such measures may also reduce the high risk of antibiotic resistance transfer from animals to humans (Heuer et al. 2006; Smith et al. 2005). Antibiotics were phased out as growth promoters in 1986 in Sweden, followed by Denmark in the late 1990s, and subsequently the European Union. The action in Denmark was stimulated by the identified linkage between avoparcin use in broiler chickens and vancomycin-resistant enterococcal (VRE) infections in humans (Bates 1997). Overall, a dramatic decline in the total use of veterinary antibiotics was achieved in Denmark: from ≥ 200 metric tons in 1994 to around 70 metric tons in 1999 (DANMAP 2010). However, “therapeutic” use of antibiotics in Danish pigs slowly doubled over a 10-year period, but was curtailed by about 25% after stricter monitoring and enforcement against illegal use in 2010–2011 (Aarestrup 2012). Banning subtherapeutic use of antibiotics in Denmark led to marked reductions of antibiotic resistance among fecal enterococci in the animal populations (Aarestrup et al. 2001), demonstrating that it is indeed possible to reverse the occurrence of antibiotic resistance among a national population of food animals through regulations restricting antibiotic use. Multidrug resistance rates of *Enterococcus faecium* in U.S. poultry have also been observed to decline—from 84% to 17%—following a conversion to organic feed (Sapkota et al. 2011). However, initial sharp decreases can taper off, with an estimated 25 years required for vancomycin-resistant enterococci to fully dissipate (Johnsen et al. 2011). Monitoring for response of resistance carriage in humans has not revealed obvious reductions, but significant confounds are related to international travel and consumption of imported meat that may carry higher loads of resistant bacteria (Hammerum et al. 2007). Aarestrup (2012) noted the need for improved human monitoring data. Correlations have been identified between antibiotic use and sulfonamide and tetracycline ARG abundance in cattle waste lagoons in the United States (McKinney et al. 2010) and in Dutch soil (Knapp et al. 2011), supporting the relationship between antibiotic use and environmental reservoirs of resistance.

*Maintaining good animal health.* Keeping animals healthy is an important way of reducing the usage of antibiotics. Best management practices, such as low animal density and improved nutritional programs, can be developed and adopted to control infectious diseases on farms. In a recent study of antibiotic amendment in dairy calf milk replacer, subtherapeutic antibiotics provided no additional health benefit when the calves were provided a high level of nutrition (Thames et al. 2012). In contrast, Quigley and Drew (2000) observed that calves experienced greater incidence of illness when antibiotics were not supplemented, but the calves in their study received a reduced nutritional intake. Knowledgeable animal husbandry is cited as the most important factor in reducing antibiotic use (van de Weerd et al. 2009).

*Alternatives to antibiotics.* Metals [such as copper (Cu), zinc (Zn), and arsenic (As)] are commonly used in animal feeds as alternatives to antibiotics (Bolan et al. 2004). Because antibiotic resistance can be co-selected by metals (Berg et al. 2010; Knapp et al. 2011; Scientific Committee on Emerging and Newly Identified Health Risks 2009), it is apparent that replacement of antibiotics with metals could actually make antibiotic resistance worse. Further, metals (notably Cu) can accumulate in agricultural soils (Bolan et al. 2004; Gräber et al. 2005), and thus serve as even stronger long-term selective agents for antibiotic resistance in manure-amended soils than do antibiotic residues, which are more prone to degradation and/or sequestration (Chee-Sanford et al. 2009). Other alternatives, such as herbal materials, may be worth pursuing (Hanczakowska and Szewczyk 2007); however, they should also be evaluated for the potential to select for antibiotic resistance because many of them exert antimicrobial activity. Increased availability of inexpensive, readily deliverable (ideally orally) vaccines that target major bacterial pathogens of animals, poultry, and fish would be very desirable.

Although antibiotic resistance may decline after relaxation of selection pressures, low yet detectable levels of resistance determinants are likely to persist for decades because of the low fitness costs associated with many antibiotic resistance mechanisms (Andersson and Hughes 2010; Johnsen et al. 2011). McKinney et al. (2010) reported that sulfonamide and tetracycline ARGs were only slightly less abundant in lagoons receiving organic versus conventional dairy waste. Both organic and conventional cattle lagoon water have been reported to contain average *tet*(W) (tetracycline resistance) and *sul*1 (sulfonamide resistance) levels about three orders of magnitude greater than those in “pristine” background river sediment in this same region (Pruden et al. 2012). This indicates that even under minimal antibiotic use conditions (organic), there is a potential for release of ARGs. Therefore, ideal management practices will aim to control the flow of genetic elements from animal manure to aquatic systems.

*Management of manure containing antibiotics.* Composting eliminates on average 50–70% of some antibiotics (Sharma et al. 2009; Storteboom et al. 2007; Wang et al. 2012; Wu et al. 2010). Antibiotic degradation is suspected to primarily occur only during the thermophilic phase over the first 2 weeks, and efficiency depends on both duration and temperature. Storteboom et al. (2007) observed that watering, aeration, and turning of compost offered some advantage to accelerating antibiotic decay of chlortetracycline, monensin, and tylosin, but even simple storage of manure stockpiles resulted in significant antibiotic degradation. Digestion of livestock waste can also treat antibiotic residues; 5-week fermentation effectively removed most sulfonamides and trimethoprim (Mohring et al. 2009), whereas sulfamethoxazole and oxytetracycline were reduced more effectively under aerobic than anaerobic incubation of dairy lagoon water (Pei et al. 2007).

*Biological treatment of ARGs in manure.* Response of ARGs to biological treatments, such as lagoons and composting, varies because of the complex microbial ecology involved. Composting and manure storage resulted in up to 100-fold reduction of tetracycline ARGs, but *tet*(O) increased when horse manure was composted, even in the absence of measurable antibiotics (Storteboom et al. 2007). Persistence of ARBs (such as *Escherichia coli*) and ARGs [*tet* and *erm* (erythromycin resistance methylase)] has been observed after composting (Sharma et al. 2009), and ARGs can persist even in the absence of selection pressure (Johnsen et al. 2011). McKinney et al. (2010) observed up to 10-fold reduction of *tet* ARGs across six anaerobic livestock lagoons monitored, but *sul* ARGs tended to increase with treatment time. Other researchers have reported lagoon treatment to be less effective than composting (Wang et al. 2012). A recent laboratory study with an agricultural *E. coli* strain suggested that anaerobic treatment may be a promising way to impose a high metabolic burden on bacteria and thus limit their capability to engage in horizontal gene transfer (Rysz et al. 2013).

*Containment of ARGs in manure.* Containment of animal wastes is a practical strategy with other advantages of nutrient management and protection of soil and water quality. Containment strategies include prevention of lagoon spills and seepage, control of surface runoff, and limiting sediment erosion and transport from animal farms. Surface runoff can be limited by improved manure collection and increased storage capacity, allowing for manure application to land only when crop demands for water and nutrients are high. Long-term manure storage offers benefits in terms of containment and can result in reduced prevalence of tetracycline residues and tetracycline-resistant bacteria (Chee-Sanford et al. 2009). Manure separation technologies act to concentrate solids from manure slurries through processes such as screening, filtration, or sedimentation and may also provide an avenue to mitigate the release of antibiotic residues and ARGs. Benefits of manure separation include reduced nutrient content, prolonged storage potential, improved biological treatment, and minimization of odors.

*Potential synergies with alternative energy or policy needs.* On-farm methanogenic biogas facilities may provide added incentive for improved waste treatment (Mohring et al. 2009). The increased intensification and geographical concentration of livestock production facilities further solidifies incentives to consider novel manure management technologies (Steinfeld et al. 2006).

At a policy level, standards on concentrations of antibiotics in animal manures for land application should be established and monitored. Using animal manures as organic fertilizer also reduces the runoff from animal farms and the risk of lagoon spills and seepages while allowing nutrient recovery. Enacting controls on manure management is challenging because it requires agreement, cooperation, and enforcement among a large number of stakeholders.

### Domestic, Hospital, and Industrial Wastewater Treatment

*Need for sanitation and sewage treatment in the developing world.* The WHO (2012b) estimated that globally 2.6 billion people lack access to basic sanitation, which likely results in direct releases of ARBs and pathogens into the environment and ambient waters. Thus, basic hygiene is likely a critical step to mitigating the spread of resistance. Of recent concern is the detection of the *NDM-1* gene in polluted surface waters and chlorinated tap water in India (Walsh et al. 2011). *NDM-1* provides bacteria with resistance to a large number of antibiotics; it is highly mobile and is found in multiple waterborne pathogens, including *Vibrio cholera* (Walsh et al. 2011) and *E. coli* (Kumarasamy et al. 2010).

*Fate of antibiotics in wastewater treatment plants (WWTPs*). Sewage collection and treatment serves an essential role in the protection of human and environmental health. These systems are designed to remove conventional pollutants, including suspended solids, nutrients (nitrogen and sometimes phosphorus), organic matter, and, to some extent, pathogens. Traditional WWTPs are not designed for the removal of antibiotics or ARGs.

Antibiotic residues from different sources (household, pharmaceutical industry, and hospital) enter into municipal sewage along with other co-selecting factors, such as metals and surfactants. At least 56 antibiotics belonging to six different classes have been widely detected at nanogram-per-liter to microgram-per-liter levels in sewage of East Asia, North America, Europe, and Australia (Zhang and Li 2011). Removal pathways include adsorption, biodegradation, disinfection, and membrane separation (Zhang and Li 2011). Other pathways, such as hydrolysis, photolysis, and volatilization, also contribute to removal (Zhang and Li 2011), depending on antibiotic properties. For example, tetracyclines are removed mainly by adsorption onto the biomass flocs; beta-lactams are largely degraded by hydrolysis reactions driven by bacteria or physical chemical processes; and erythromycin and ciprofloxacin are recalcitrant toward biodegradation in activated sludge (Li and Zhang 2010).

Antibiotics pose a special problem for wastewater treatment because they may impose selective pressure. The same mechanisms that degrade antibiotics can also enable resistance and be selectively enriched (e.g., beta-lactam degradation) (Baquero et al. 1998). Clearly, the role of antibiotics as selective agents in WWTPs is complex. In a recent study of a domestic WWTP, Gao et al. (2012) observed a correlation between certain sulfonamide ARGs and sulfonamide antibiotics, but no correlation between tetracycline ARGs and corresponding antibiotics. At present, the possible role of antibiotics as selective agents in municipal WWTPs remains unclear.

*Fate of ARBs and ARGs in WWTPs.* WWTPs receive direct input of resistant fecal and commensal bacteria from patients prescribed antibiotics. Most recently, methicillin-resistant *Staphylococcus aureus* (MRSA) was detected in the effluent of four U.S. WWTPs (Goldstein et al. 2012), and bacteria resistant to clinically important antibiotics, including ciprofloxacin and vancomycin, have been found in the activated sludge (Nagulapally et al. 2009). ARBs and ARGs may either decrease (i.e., via death and decay) or increase (i.e., via horizontal gene transfer and/or selective enrichment) through the treatment process. The most direct route of removal of both ARBs and ARGs is via solids separation, such as sedimentation. However, subsequent biological treatment steps may result in selective increase of ARBs (Zhang et al. 2009). Using plasmid metagenomic analysis, Szczepanowski et al. (2009) reported evidence that new ARGs found in clinical bacterial isolates had resulted from exchange with wastewater bacteria. In addition, WWTPs appear to possess the ideal mix of conditions to foster horizontal gene transfer and development of multidrug-resistant bacteria (Schlüter et al. 2007). ARGs persist in effluents of a variety of full-scale WWTPs at levels well above those typical of aquatic environments, even after disinfection (Auerbach et al. 2007). ARGs have even been observed to break through relatively advanced WWTPs that use mixed-media filtration, and persist at detectable levels in surface water receiving the discharge (LaPara et al. 2011). Other researchers have observed ARGs from industrial and municipal WWTP sources to persist in river sediment (Kristiansson et al. 2011; Storteboom et al. 2010).

*WWTPs as critical control points.* WWTPs may represent a critical node for control of the global spread of antibiotic resistance. Thermophilic anaerobic sludge digestion appears particularly promising and may achieve superior ARG removal relative to mesophilic digestion, potentially because of the much narrower host ecology of the microorganisms (Diehl and LaPara 2010; Ma et al. 2011). More advanced treatment technologies (e.g., membrane separation) could be applied to retain bacterial cells, including their genetic material (Riquelme Breazeal et al. 2013). In addition, ozone has been proposed to disinfect ARBs and destroy ARGs (Dodd 2012). Because costs of advanced treatments will be significant, an ideal place to start may be to consider ARGs alongside other issues of concern if upgrades are already planned.

*Wastewater reuse.* Wastewater reuse is becoming a worldwide strategy for water sustainability. However, it is critical to carefully evaluate the application of reclaimed water and establish proper safeguards in order to avoid unintended consequences. It is common practice to employ different treatment levels for different purposes (e.g., bathing vs. toilet flushing or irrigation). Wastewater is commonly disinfected via ultraviolet radiation or chlorination, which may kill resistant bacteria, but ARGs are more recalcitrant (Auerbach et al. 2007; Kim et al. 2010; McKinney and Pruden 2012; Munir et al. 2011). Li and Zhang (2012) reported that chlorination reduces several antibiotics, including ampicillin, chlortetracycline, sulfamethoxazole, sulfadiazene, ofloxacin, and trimethoprim. Ozonation has been reported to efficiently reduce a broad range of antibiotics and their active metabolites (Dodd et al. 2010).

*Sludge/biosolids and other solid wastes.* Land application of sludge/biosolids from WWTPs, another means of resource recovery, also serves a second purpose: disposal of a costly treatment by-product. However, ARBs and ARGs are known to be present in biosolids (Brooks et al. 2007; Munir et al. 2011). Research suggests that culturable heterotrophic ARBs attenuate quickly after amendment to soil (Brooks et al. 2007), but studies employing culture-independent techniques indicate otherwise. For example, in a recent study comparing land application of manure versus biosolids, Munir and Xagoraraki (2011) found elevated levels of tetracycline and sulfonamide ARGs in soils amended with biosolids during the 4-month monitoring period. Interestingly, the effect was more strongly driven by soil characteristics than by the source. Munir et al. (2011) also noted that among five U.S. WWTPs, the loading rate (mass × concentration) of tetracycline and sulfonamide ARBs and ARGs produced in biosolids was ~ 1,000 times higher than that in aqueous WWTP effluent.

Antibiotics are prevalent in biosolids and in household and hospital solid wastes. In biosolids from East Asia, North America, and Europe, 17 antibiotics from five classes were detected at levels of micrograms per kilogram to milligrams per kilogram (dry sludge weight) (Zhang and Li 2011). Incineration is a zero-risk solution with regard to reduction of antibiotics, ARBs, and ARGs, although there are trade-offs with air quality and cost of alternative fertilizers. If used appropriately, incineration may provide a source of alternative energy. Landfills still pose some risks because leachates may pollute groundwater and surface water, and they are commonly redirected to a municipal WWTP (Renou et al. 2008). In Sweden, only 1% of household waste was deposited in landfills in 2010, whereas 99% was either incinerated or recycled (Naturvårdsverket 2012).

*Hospital and industrial waste treatment: hot spots for antibiotic resistance.* Resistant microbes have the potential to rapidly spread from one corner of the world across the entire planet (Walsh et al. 2011); thus, managing “hot spots,” such as hospitals and drug manufacturers, is of high concern (Kovalova et al. 2012). Hospitals are of interest for targeted pretreatment systems, such as membrane bioreactors, that can partially remove antibiotics and other drugs, as well as ARBs, before discharging into public sewer systems (Kovalova et al. 2012). Recently, a multiple-criteria decision analysis of options and motivation for removing pharmaceuticals from hospital wastewater in Switzerland indicated remarkably high acceptance of this approach across multiple stakeholders (Lienert et al. 2011).

Manufacturing sites were identified as potential hot spots for antibiotic-resistance development only a few years ago, with levels reaching milligram-per-liter concentrations in several cases. Larsson et al. (2007) found exceptionally high levels of fluoroquinolones in the treated effluent of a WWTP serving approximately 90 generic drug manufacturers in India. In the same area, severe antibiotic contamination was found in the local surface, ground, and drinking waters (Fick et al. 2009), and ARGs and associated mobile genetic elements were markedly increased downstream (Kristiansson et al. 2011). Studies from China showed releases of therapeutic levels of oxytetracycline and penicillin downstream from a factory, with increased resistance rates (Li et al. 2009, 2010). Sim et al. (2011) reported lincomycin concentrations up to 44 mg/L in the effluent from a Korean factory, and a Croatian study reported releases of sulphonamides at concentrations up to milligrams per liter (Babić et al. 2007). One factory annually contributed about 2,000 kg of antibiotic to a WWTP in Oslo, Norway; this was considerably more than the amount of any active pharmaceutical ingredient (API) studied that originated from usage and excretion (Thomas et al. 2007). A crucial question is whether these are exceptions or the norm. This question is difficult to evaluate because publically available data on antibiotic emissions from drug manufacturing are still highly fragmented.

Some industries treat their own wastes from its generation through to discharge, while others discharge to a third party WWTP with or without pretreatment (e.g., pH adjustment, chelation, precipitation). Therefore, the level of control and accountability differs. Production cycles at pharmaceutical manufacturing sites are highly variable, and many drugs are produced in a batch-wise manner; thus, effluent composition can vary drastically over time. This variation in composition requires distinct treatment relative to domestic WWTPs, which are designed to receive stable loadings. Thus, WWTPs that receive wastes from drug manufacturers will benefit from requiring pretreatment or establishing limits to antibiotic discharge.

Variable waste streams typical of industrial production will likely require a range of treatment technologies. A major challenge is that the high antibiotic concentrations in industrial WWTPs inevitably will exert strong selection for ARBs. For this reason, activated sludge is not recommended for highly antibiotic-contaminated waste streams because of the high density of microbial populations. If biological treatment is unavoidable, bacteria from the treatment process must be eliminated before discharge. We discourage seeding biological treatment systems with microbes originating from human feces, as well as land-application of residual biosolids from hot-spot sources.

Several policy measures could provide benefits for curtailing the spread of antimicrobial resistance from hot spots. First, the industry itself could take a leading role in developing voluntary standards for pharmaceutical wastes containing APIs (Murray-Smith et al. 2012). It may be worthwhile to impose more restrictions on synthetic antibiotics and those that persist in the environment (e.g., fluoroquinolones). Second, greater transparency through the supply chain is urgently needed in order to indicate where human drugs are coming from and where they are going (Larsson 2010; Larsson and Fick 2009). Third, national purchasers of medicines could aim to take greater responsibility of the issue [Swedish Environmental Management Council (SEMC) 2011]. Action in this area is critical because many governments are focusing on cost as the primary driver of policy decisions. Finally, extension of good manufacturing practices to include environmental considerations could be of benefit [Medical Products Agency (MPA) 2011].

### Aquaculture Management Options

Infectious disease outbreaks among aquaculture stock species are of fundamental concern because of both loss of stock and detriment to animal welfare. Aquaculture is increasing worldwide (Bostock et al. 2010), which is likely to increase the disease risk. Because the primary motivation of antibiotic use in aquaculture is to protect against the devastation of stock illness and loss, promoting a healthy fish stock is the ideal route for minimizing antibiotic use. In some countries (e.g., in North America and in Europe), licensing and regulation of the use of antimicrobial agents in aquaculture is strictly enforced and guided by veterinary professionals. However, a large proportion of the global aquaculture production takes place in countries with few regulations and limited enforcement (FAO/OIE/WHO 2006).

For economic reasons, quinolones, sulfonamides, and tetracyclines are the most popular antibiotics in aquaculture, although others such as macrolides and beta-lactams are also occasionally used (FAO/OIE/WHO 2006). Most fish species cultivated in aquaculture are poikilothermic and are adapted to lower temperatures (Heuer et al. 2009); however, some zoonotic fish bacteria, such as *Aeromonas, Salmonella*, and *Mycobacterium,* can also infect humans and carry ARGs (Weir et al. 2012). Bacteria such as *E. coli* can be present in water and on harvested fish, especially when animal or human waste is added, as is the case in integrated production systems. *E. coli* is the most common bacterial human pathogen, and exposure to antibiotics in the aquaculture environment may stimulate elevated resistance. Approximately 20 years after industrial aquaculture had begun, evidence emerged that ARGs were transferred between aquatic bacteria that are pathogenic to both fish and humans (Cabello 2006; Ryu et al. 2012). In the case of cultured shellfish, deadly pathogens, such as *Vibrio* and *Salmonella,* may acquire resistance via horizontal transfer. For example, the fish pathogens *Vibrio* and *Lactococcus* transferred tetracycline ARGs to human *E. coli* and *Enterococcus faecalis* (Neela et al. 2009). A joint FAO/OIE/WHO expert consultation on *Antimicrobial Use in Aquaculture and Antimicrobial Resistance* (FAO/OIE/WHO 2006) and Cabello (2006) concluded that public health hazards related to antibiotic use in aquaculture include the development and spread of ARBs and ARGs, as well as the occurrence of antibiotic residues in aquaculture products.

In some developed countries, newly introduced vaccines (Sommerset et al. 2005) and well-equipped facilities have helped alleviate the need for antibiotics. This is exemplified by a 99% reduction in the use of antimicrobial agents in Norwegian salmon and rainbow trout aquaculture from 1987 to 2007, despite a massive increase in fish production (Heuer et al. 2009). However, developing countries, especially Asian countries where the majority of aquaculture production occurs, suffer from proliferation of ARBs stimulated by aquaculture management system practices and each farmer’s lifestyle (Heuer et al. 2009). Integrated farming of animal–fish–vegetable, in which antibiotics are used for animal husbandry and animal waste is directly released to aquaculture ponds and applied to rice/vegetable fields, is common in Southeast Asia. This practice causes direct antibiotic contamination and can select for ARBs (Suzuki and Hoa 2012). However, this practice is traditional and thus not straightforward to eradicate. Therefore, international monitoring will be especially important for products from integrated ponds.

Rearing methods for fish are roughly divided into land-based pond and marine pen culture. One of the fundamental ways to abate diseases is to reduce the animal density, which can reduce physical contact and fighting. Preventing invasion of wild fish into pens is also crucial because exchange of pathogenic fish bacteria between wild and cultured fish is a suspected mechanism of spreading ARGs (Grigorakis and Rigos 2011). It is also important to avoid overuse of feed: Excess feed will settle, augment the bacterial reservoir, and contribute to an unhealthy, eutrophic environment.

Fish feed can also serve as a direct source of ARBs and ARGs. Minced raw fish meat commonly used for feed can contain a diverse microbiota, as well as mixtures of other materials such as soybean and vegetable oil. Dry pelleted food may offer some advantages and is gaining popularity, having been used exclusively in salmon and trout aquaculture since the 1970s (Takeda 2010). Most non-spore bacteria will be sterilized in the heating process of feed manufacturing; however, residual gram-positive spores and their DNA have introduced ARGs in marine environments (Rahman et al. 2008).

Aquaculture workers in areas with intensive use of antibiotics are directly exposed to both antibiotics and ARBs, and are therefore likely to be at increased risk for antibiotic-resistant zoonotic and foodborne infections. We believe that the greatest potential risk to the broader public is the development of a reservoir of transferable ARGs in aquatic bacteria that can be disseminated by horizontal gene transfer to other bacteria and ultimately to human pathogens. However, a quantitative risk assessment on antibiotic resistance in aquaculture is difficult to perform because of a lack of data and the complex pathways of gene flow among various aquatic species and environmental compartments. Programs to monitor antibiotic use and ARBs from farm-raised aquatic animals and their surroundings should be implemented, and national databases are needed to provide baseline information and facilitate communication (FAO/OIE/WHO 2006).

Finally, aquaculture exemplifies the international transport risk of ARGs. In 2009, China produced 62.5% of the global harvest of fish, crustaceans, and molluscs (34.8 million metric tons). Five other countries produced > 1 million metric tons in the same year (Bostock et al. 2010). Developed countries import a significant portion of the harvest, accounting for 76.8% of total fisheries imports (in value), with the European Union accounting for 40.8% and the United States and Japan together accounting for 27.2% of the total. One approach to limiting international pathways may be to monitor antibiotic residues at customs. Although there are innumerable ARGs in environment, at least those with high clinical relevance, such as *mec*A, extended spectrum beta-lactamases, and *NDM-1*, could also be monitored.

## Strategic Implementation and Monitoring Needs

Although it is not possible to define safe exposure levels in a strict sense, the scientific community should aim to define such levels to provide regulators with a basis for defining and implementing standards. Once standards are defined, it will be possible to estimate costs associated with various mitigations. However, we must acknowledge that the uncertainty is still high regarding ultimate benefits for individual measures. On the other hand, anticipated societal costs associated with increased resistance motivate mitigations, even without conclusive evidence that their implementation will lead to less clinical treatment failures in the future. It will be extremely difficult to quantify such links all the way to clinical outcomes. Therefore, at present, efficacy of mitigation efforts can best be evaluated on the basis of surrogate measures, such as the abundance of antibiotics, ARGs, and ARBs in the environment. Routine monitoring programs are required to provide baseline data on which to contrast measurements before and after mitigation activities, as has been successfully implemented by DANMAP. Establishing and/or maintaining existing biobanks of soil and water will allow retrospective analyses. Similarly, metagenomic inventories allow retrospective *in silico* analyses of resistance factors that we are not concerned about now, but may be of concern later.

### Incentives and Risk Communication

Many stakeholders are involved in each of the above proposed management options, and understanding their various incentives is key. Generally, economic incentives are the strongest, but political or reputational incentives can also be important. Short-term costs are often a major contraincentive to invest in mitigations, whereas branding through environmental responsibility and concern over public health are general proincentives.

Economic incentives can be provided at different levels through the adjustment of business models or regulatory actions, such as increased costs or strict penalties for noncompliance. For example, current business models have not provided sufficient leverage for the pharmaceutical industry to invest in the development of new antibiotics at the necessary pace to keep up with resistance. The lack of innovation in antibiotic discovery and increased reliance on existing antibiotics have contributed to increased prevalence of resistance and the reduced efficacy of existing treatment options. There is a growing pressure for antibiotic discovery to be refined, and several incentives have recently been proposed (Laxminarayan and Powers 2011; Spellberg et al. 2012). These incentives on their own may not remove the selective pressures for resistance development; they will just provide new ones. Therefore, any new incentives need to be coupled with increased management of antibiotics.

Economic and political pressure originating from the final consumers should not be underestimated. This is a parallel mitigation path that potentially is much faster than regulatory actions. At times, activities unrelated to the issue of concern can cause incentives. For example, animal welfare concerns may result in both reduced need for antibiotics and reduced stocking densities. Regulating pollution levels of other chemicals could also indirectly result in reduced antibiotic release.

Providing information to stakeholders and policy makers is equally as important as incentives. If stakeholders are not able to estimate risks and benefits involved with taking action, they are more likely to remain passive and go on with “business as usual.” Both the scientific community and the media have a strong responsibility to promote well-balanced risk communication. Risk communication with respect to antibiotic resistance is particularly challenging. For example, some individuals could become confused and not take antibiotics when needed. Educational campaigns, such as e-Bug (European Commission on Research & Innovation 2012), work to address this problem.

### Case Study: Stakeholder Initiatives to Reduce Risks Associated with Drug Manufacturing

Recent action within this area provides a good case study of risk management in the real world. Soon after Swedish media coverage of a study on industrial antibiotic pollution in India (Larsson et al. 2007), the Swedish Association of the Pharmaceutical Industry AB (LIF), a trade organization for research-intensive pharmaceutical companies, requested that the Swedish government take action. In parallel, the organization arranged round-table discussions with politicians, the Swedish MPA, pharmaceutical industries, county councils, pharmacies, the water treatment sector, the Swedish Environmental Protection Agency, the Swedish Chemical Agency, and academia. The direct sharing of information across stakeholders with different expertise built a common platform for discussions, and was a major reason why a strong consensus was rapidly reached that mitigations were necessary. In 2009, the Swedish government formally commissioned the MPA to identify ways to reduce pollution from pharmaceutical industries on a global basis. Eight different actions were proposed, where the main path was to amend the Good Manufacturing Practice framework with environmental criteria (MPA 2009, 2011). Sweden has now brought this proposal to the European Union health ministry.

In early 2009, the Associated Press highlighted the pollution situation in India incentivising several major international companies to intensify their work with internal operations and third party supply-chains. An example of this is a proposed scheme by AstraZeneca for defining “safe” discharge limits for active pharmaceutical ingredients from manufacturing sites (Murray-Smith et al. 2012).

The SEMC and the county councils implemented new environmental procurement criteria for medicines for hospital use in 2011 (SEMC 2011). For the first time, focus is on emissions from manufacturing. No discharge limits have yet been specified, but suppliers and subcontractors have to set up monitoring programs. Well before implementation, seminars were arranged and all major medical suppliers were invited.

To create further incentives, the Swedish government has drafted a proposal regarding the national generic substitution system (Swedish Government 2013). Previously, cost reduction has been the sole driver to identify therapeutically interchangeable products that will be (partly) reimbursed by the state. If the proposal is implemented, companies would compete not only on price but also on their level of pollution control. An expected hurdle for implementation is how environmental risks associated with manufacturing should be assessed. To address this issue, a group of stakeholders initiated work in 2011 to generate a draft document on life-cycle environmental classification. In 2011, the Swedish government (2011) also adopted a “National Pharmaceutical Strategy.” Reducing environmental emissions of drugs, nationally as well as globally, was one of the major aims highlighted in this strategy. A major challenge is that the site of origin of the API is confidential. Thus, major business journals in Sweden have highlighted the need for greater transparency (Larsson 2010; Larsson and Fick 2009). Clearly, there are economic risks linked to negative media exposure, and this drives action. Along these lines, as major shareholders in the pharmaceutical industry, the Swedish Church arranged a seminar for the bank sector in late 2012 to provide guidance for how to act, as shareholders, in order to promote environmentally safe production.

## Conclusion

We identified several management options across agriculture, wastewater treatment, aquaculture, and pharmaceutical manufacturing that could aid in mitigating risks of antimicrobial resistance in the environment. Many of these are practical strategies that are economically feasible and that can be synergistically implemented with other benefits. Recent proactive measures taken in Sweden demonstrate that such actions are possible and add momentum to the development of new policies and regulations. Outreach, education, communication, monitoring, and transparency are vital for the success of management schemes for limiting the spread of antibiotic resistance via environmental pathways.
